# A Computational Analysis of Limb and Body Dimensions in *Tyrannosaurus rex* with Implications for Locomotion, Ontogeny, and Growth

**DOI:** 10.1371/journal.pone.0026037

**Published:** 2011-10-12

**Authors:** John R. Hutchinson, Karl T. Bates, Julia Molnar, Vivian Allen, Peter J. Makovicky

**Affiliations:** 1 Structure and Motion Laboratory, Department of Veterinary Basic Sciences, The Royal Veterinary College, Hatfield, Hertfordshire, United Kingdom; 2 Department of Musculoskeletal Biology, Institute of Aging and Chronic Disease, University of Liverpool, Liverpool, United Kingdom; 3 Department of Geology, Field Museum of Natural History, Chicago, Illinois, United States of America; College of the Holy Cross, United States of America

## Abstract

The large theropod dinosaur *Tyrannosaurus rex* underwent remarkable changes during its growth from <10 kg hatchlings to >6000 kg adults in <20 years. These changes raise fascinating questions about the morphological transformations involved, peak growth rates, and scaling of limb muscle sizes as well as the body's centre of mass that could have influenced ontogenetic changes of locomotion in *T. rex*. Here we address these questions using three-dimensionally scanned computer models of four large, well-preserved fossil specimens as well as a putative juvenile individual. Furthermore we quantify the variations of estimated body mass, centre of mass and segment dimensions, to characterize inaccuracies in our reconstructions. These inaccuracies include not only subjectivity but also incomplete preservation and inconsistent articulations of museum skeletons. Although those problems cause ambiguity, we conclude that adult *T. rex* had body masses around 6000–8000 kg, with the largest known specimen (“Sue”) perhaps ∼9500 kg. Our results show that during *T. rex* ontogeny, the torso became longer and heavier whereas the limbs became proportionately shorter and lighter. Our estimates of peak growth rates are about twice as rapid as previous ones but generally support previous methods, despite biases caused by the usage of scale models and equations that underestimate body masses. We tentatively infer that the hindlimb extensor muscles masses, including the large tail muscle M. caudofemoralis longus, may have decreased in their relative size as the centre of mass shifted craniodorsally during *T. rex* ontogeny. Such ontogenetic changes would have worsened any relative or absolute decline of maximal locomotor performance. Regardless, *T. rex* probably had hip and thigh muscles relatively larger than any extant animal's. Overall, the limb “antigravity” muscles may have been as large as or even larger than those of ratite birds, which themselves have the most muscular limbs of any living animal.

## Introduction

*Tyrannosaurus rex* Osborn 1906 (Dinosauria: Theropoda: Tyrannosauridae) [1] has become an exemplar taxon for palaeobiological studies because of its large body size, suite of near-complete skeletons, and popular appeal [2]. It is immediately recognizable to many people worldwide for not only its giant size but also its unusual anatomy: large head, small forelimbs, robust torso and tail, and long hindlimbs. The anatomy of its hindlimbs in particular has been the focus of comparative studies for almost a century [3]–[19].

Most recently, studies of tyrannosaur hindlimbs have emphasized biomechanical procedures to quantitatively test functional hypotheses about locomotion [20]–[28]. Furthermore as part of a renaissance in musculoskeletal biomechanics that was initiated by Alexander [9]–[10] and hastened by technological developments, tyrannosaur morphology has been a major subject for computational analyses of three-dimensional (3D) whole-body shape. In particular, studies have focused on inertial properties such as mass, centre of mass and inertia [26]–[31] that are critical for biomechanical analyses.

**Figure 1:**
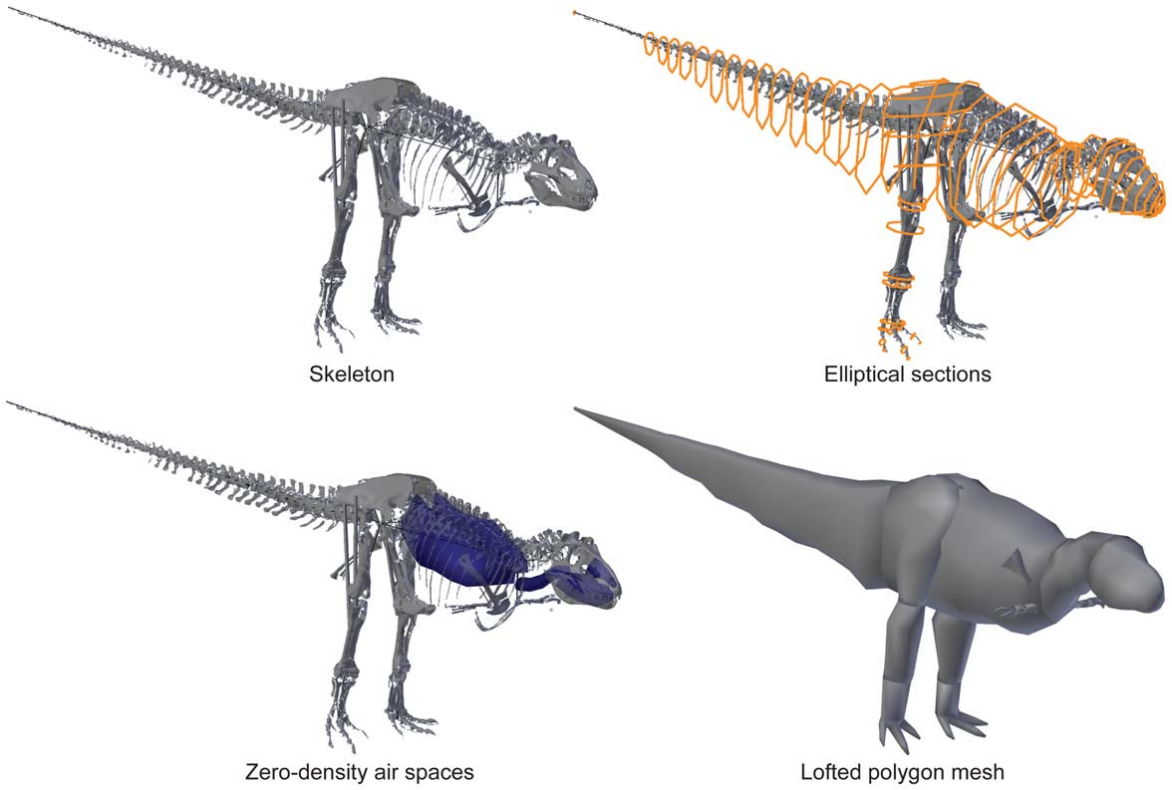
Modelling procedure, showing the Carnegie specimen. From left to right, top to bottom these show the scanned, reconstructed, and straightened skeleton; the skeleton with elliptical hoops that define fleshy boundaries; the air spaces representing pharynx, sinuses, lungs and other airways including air sacs; and the final meshed reconstruction used for mass and COM estimates.

The use of computer models to make these estimates of inertial properties avoids some major problems of alternative approaches, such as use of scaling equations (e.g., [32]–[33]). Such models have the advantage of producing inertial property estimates specific to the taxon (and specimen) of interest and its entire anatomy. The accuracy of these estimates is presumably no worse than that of alternative methods and quite possibly better, but still has a substantial margin of error of 50% or more (e.g., [31]), which always demands careful consideration. Here our primary aim is to use three-dimensional (3D) scanning of a putative juvenile *T. rex* specimen (see below and [34]) as well as four of the best-preserved and most well known adult specimens (Table 1); including the famously well-preserved skeleton “Sue” (FMNH PR 2081); to estimate individual and ontogenetic variation in limb and body dimensions in *T. rex* (Figure 1) and their repercussions for tyrannosaur biology. As part of this aim, we seek to further understand the degree of errors intrinsic to such 3D reconstructions; as well as alternative methods such as scaling equations, following up on previous studies [26]–[31], [35].

**Table 1:** Tyrannosaur specimens used in this study, with specimen numbers and colloquial names given.

Specimen	% Bones	Scanning
CM 9380 "Carnegie"	11	LiDAR scan of mounted specimen
FMNH PR 2081 "Sue"	73	LiDAR scan of mounted specimen, CTs of limb casts
BHI 3033 "Stan"	63	LiDAR scan of cast
MOR 555 "MOR"	49	LiDAR scan of cast; limb bones point digitized at MOR
BMR P2002.4.1 "Jane"	52	LiDAR scan of cast

Bones” indicates the estimated percentage of bones (by number) preserved, from N Larson (2008).

Body mass is a key parameter for understanding the biology of any animal, let alone taxa that represent physical extremes such as *Tyrannosaurus rex* and other large dinosaurs. Most studies that use body mass estimates for dinosaurs rely on equations that are extrapolated from multi-taxon datasets of presumed adult individuals [32], [36]. These approaches approximate an average model value based on the sampled taxa and thus minimize taxonomic differences in body shape and volume. They also do not address individual and ontogenetic variation, which is critical because intraspecific scaling patterns do not follow interspecific scaling in non-avian dinosaurs [37]. Studies of dinosaurs that incorporate ontogenetic series [38]–[41] often utilize Developmental Mass Extrapolation (DME) [42] to estimate mass for immature specimens using the cube of femoral length and an assumption of isometric scaling. While this method derives support from observations on scaling in extant archosaurs, it remains untested for fossil taxa. The application of advanced 3D scanning equipment in palaeontology allows estimation of body mass for individual specimens, and thereby a potentially more accurate understanding of both ontogenetic changes in body mass as well as individual variation in this critical parameter. Such insights should increase the precision in analyses of dinosaurian biology that employ body mass estimates and allow for independent evaluation of the DME approach. Our study's second aim is to apply these techniques to *Tyrannosaurus* to revisit estimates of its growth rate and the reliability of DME.

The remarkable limb and body proportions of tyrannosaurs changed appreciably as the animals grew from relatively more slender and long-legged (i.e., cursorial; [43], [44]) juveniles into more robust adults [5], [17]. A similar pattern seems to hold for other lineages of giant theropods (e.g., Allosauroidea: [45]). *Tyrannosaurus rex* is unusual relative to other dinosaurs in that its femur scales according to a static stress model (i.e., length scales half as fast as diameter) through ontogeny, the only one of 24 examined non-avian dinosaurs species to unambiguously exhibit that growth pattern [37]. Tyrannosaur growth itself is fascinating from a biomechanical perspective, because *T. rex* took less than 20 years to develop from <10kg hatchlings into >6000 kg giants [39], [46]. The fast growth of larger tyrannosaurs should have caused them to face rapidly shifting size-related biomechanical constraints involving ontogenetic allometry [31], [47]–[55]. Hutchinson and colleagues [20], [21], [25] used an integration of limb musculoskeletal biomechanics and estimates of inertial properties to assess Currie's [17], [56] hypothesis that young tyrannosaurs were at least relatively, if not absolutely, more athletic than larger, older tyrannosaurs and thus that tyrannosaur ecology changed drastically across ontogeny (as in some other species; e.g., *Alligator* [57]). Juvenile *Tyrannosaurus* specimens were unknown at the time so they used a juvenile *Gorgosaurus libratus* specimen for comparison, and found support for Currie's [17], [56] inference.

However, a reasonably complete juvenile specimen probably pertaining to *Tyrannosaurus rex* has recently been discovered (BMR P2002.4.1; [58]–[59]). This specimen (still awaiting formal description) was originally referred to the small Maastrichtian tyrannosaurid *Nanotyrannus* [60], which many workers consider to represent juvenile individuals of *T. rex* [16], [61]–[62]. Discovery of this specimen, combined with past studies of tyrannosaur ontogeny and individual variation [16], [61]–[66], opens new potential to quantify ontogenetic changes of 3D body proportions in *T. rex* using modern computational tools, and to compare these results to data from previous studies of *T. rex* and other taxa (extant and extinct). This would illuminate more precise details of how the biology of this famous carnivore may have changed during its lifetime or otherwise varied between individuals, related to the second aim of our study noted above.

Our study's third aim is to re-examine locomotor ontogeny in *Tyrannosaurus*. We do this first by estimating hindlimb muscle masses. The sizes of those muscles are pivotal information for reconstructing how large and small tyrannosaurs moved [20], [21]. The hindlimb extensor (antigravity) muscles would have been critical determinants of maximal locomotor performance in tyrannosaurs [20]–[22], [24], [25], [67], [68]. This notion fits prevailing evidence for speed limitations in other bipeds (reviewed in [21], [67]; also [69]–[71]). Hutchinson et al. [28] provided simple geometric estimates of how much muscle mass different limb segments could contain (ranging from 2–10% body mass from the shank to the thigh, depending on assumptions about the bulk of soft tissues). An improved reconstruction methodology for estimating the mass of M. caudofemoralis, an important hip extensor muscle group, was presented by Persons and Currie [19].

We develop two methods here for analyzing limb muscle masses. We use a methodology (detailed below) similar to that of Persons and Currie [19] to investigate tyrannosaur ontogenetic changes in the mass of M. caudofemoralis longus, certainly the largest extensor muscle in the hindlimb. As a simple but critical validation step, we apply the same method blindly to a crocodile skeleton and compare the muscle mass estimate to that obtained via dissection of the same specimen. A second approach that we implement here is to subtract actual bone volumes from estimated segment volumes (as suggested by [30]) to estimate soft tissue volume, then use those volumes to estimate how large the extensor muscles acting about each hindlimb joint may have been.

These methods are then used to test our hypothesis that juvenile tyrannosaurids, using the “Jane” specimen as an exemplar, had relatively larger extensor (e.g., antigravity) muscles than adults. This hypothesis agrees with observations of the proportionately smaller heads, longer limbs, more lightly-built presacral regions and possibly shorter tails in juvenile tyrannosaurs [5], [13], [17], [66]. It also fits well with speculations about their increased relative locomotor performance and differing predatory ecologies [21], [39], [56] as well as general trends in the ontogenetic scaling of extant taxa (references cited above). Contrary to this hypothesis, Persons and Currie [19] concluded the opposite; that leg muscles scaled with positive ontogenetic allometry in tyrannosaurs to mitigate the relative decline in locomotor muscle force and power output as body size increases interspecifically (e.g., [47]–[55], [72]), although other species may do the opposite during ontogeny (e.g., [49]–[55]). Alternatively, it is also important to consider that perhaps unknown parameters are too numerous to falsify either hypothesis or the null hypothesis of no difference in leg muscle mass across ontogeny in extinct dinosaurs.

Also pertaining to the third aim of our study (reconstructing locomotor ontogeny), we focus on a second hypothesis. We propose that the aforementioned changes of body proportions in tyrannosaur ontogeny would have shifted the centre of mass (COM) cranially (and dorsally). Such a shift would have further worsened locomotor performance because of increasing demands placed on limb muscles by lowered effective mechanical advantage in the hindlimbs [20], [21], [28], [47]–[49]. Few studies have examined ontogenetic changes of COM (or inertia) in any taxa, extant or extinct, so this is a novel feature of our study. Initial examinations of extant crocodiles (*Crocodylus johnstoni*) and junglefowl *Gallus gallus* (ancestral wild chickens) indicate that they may shift their COMs craniodorsally during ontogeny [31], as we propose for tyrannosaurs, which may thus be a common (or even homologous) archosaurian pattern. Alternatively, the COM might maintain a conservative position or even shift caudally, potentially forestalling decreases in running performance. By combining our estimates of hindlimb extensor muscle masses with COM position we will re-evaluate previous estimates of locomotor ontogeny (and locomotor abilities at adult sizes) in tyrannosaurs while still considering the ambiguities inherent to these estimates.

## Results

We begin by outlining our results for models of the skeletons alone, which we expect to have the highest precision (adding flesh to the skeletons inevitably increases subjective errors). We then compare the masses of individual fleshed-out body segments among our five main specimens. Third, we examine mass and COM differences for the whole body models (and sensitivity analysis thereof) for all five specimens. Fourth, we detail our results for key extensor muscle mass estimations for each specimen. Finally, we present revised growth rate estimates for *Tyrannosaurus* and compare these with DME methods.

### Skeletal dimensions

**Figure 2:**
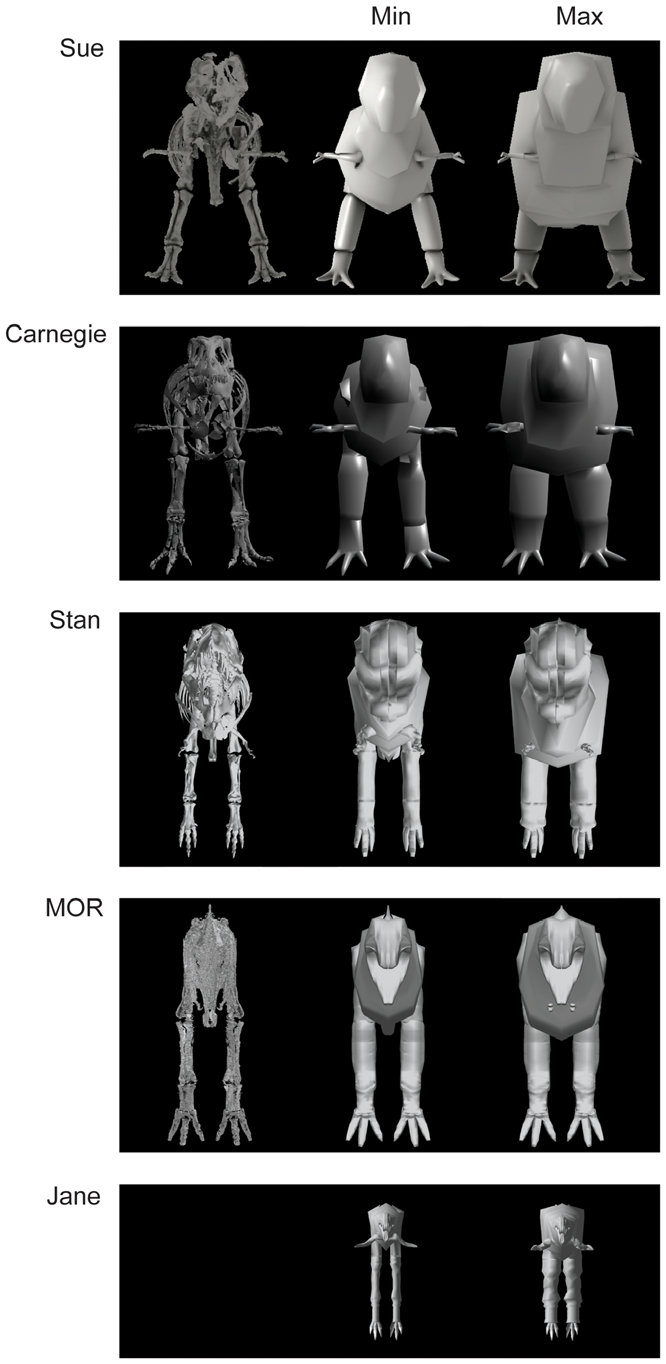
Models: cranial view. From left to right for each specimen: 3D scan of skeleton (not shown for Jane due to copyright issues), minimal model, and maximal model. Not to scale.

**Figure 3:**
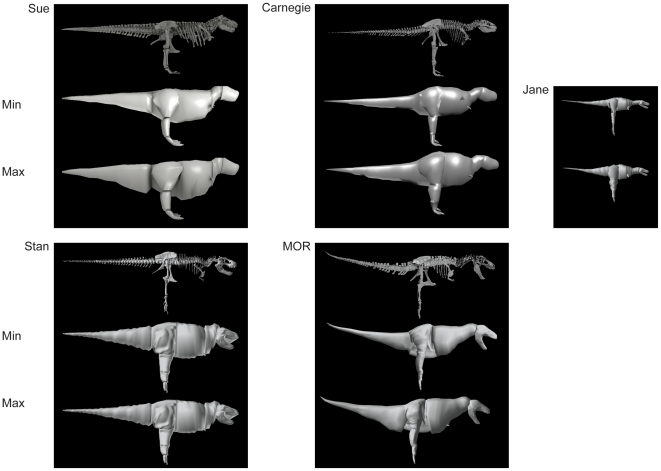
Models: right lateral view. See Figure 2, but skeleton scans/models are ordered from top to bottom.

**Figure 4:**
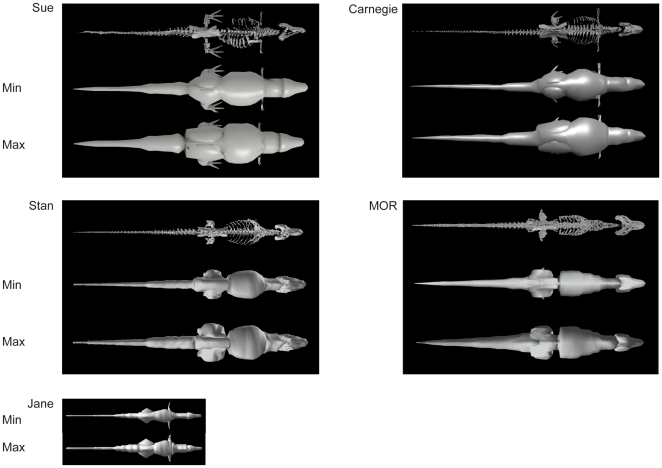
Models: dorsal view. See Figures 2,3.

The estimated volumes of each major pelvic limb bone (or set thereof) are shown in Table 2 with additional data on femur/tibiotarsus volume (as a gauge of proximal vs. distal limb size as well as preservation quality or other biases). Table 3 shows skeleton-only measurements of femur length, total head-tail tip body length, gleno-acetabular distance (GAD; between the approximate centres of those joints)/body length, limb length, and tail length (from the first free caudal vertebra)/body length. The bone volumes of our four adult tyrannosaur specimens are roughly similar. The largest specimen (Sue) has larger absolute bone volumes than all but the Carnegie specimen's, whose dimensions are partly inflated (especially for the lower limb) by the presence of a supportive metal mounting framework (Figs. 2, 3, 4) that was too tightly integrated with the bones to easily be separated. However, because the measurements for Sue were derived from CT rather than coarser-resolution optical scans, they also represent the most accurate volume measurements in the dataset. The MOR specimen exhibits a disproportionately large tibiotarsus and small femur. The latter could be partly attributable to crushing ([11]; although Hutchinson et al. [22] corrected for much of this) whereas the cause of the former difference is unclear but could easily be due to scanning methodologies and preservational distortion. Hence all values must be viewed with some caution and not read literally. The “Jane” specimen's bone volumes are around one-tenth to one-fifth those of the adult specimens. Figures 2, 3, 4 show the 3D scans of the skeletons used.

**Table 2:** Bone dimensions for the five tyrannosaur specimens.

Specimen	Segment	Volume (m^3^)	Femur/ Tibiotarsus Volume (m^3^)
Carnegie	Femur	0.0430	0.98
	Tibiotarsus	0.0438	
	Tarsomet	0.0176	
Sue	Femur	0.0440	1.39
	Tibiotarsus	0.0316	
	Tarsomet	0.0137	
Stan	Femur	0.0340	1.43
	Tibiotarsus	0.0238	
	Tarsomet	0.0140	
MOR	Femur	0.0301	1.05
	Tibiotarsus	0.0288	
	Tarsomet	0.0139	
Jane	Femur	0.00600	1.39
	Tibiotarsus	0.00433	
	Tarsomet	0.00156	

The “tibiotarsus” includes tibia, fibula and proximal tarsals. The “tarsomet” includes all mounted metatarsals, except in the case of the Sue and Carnegie specimen scans which lack the small first digit and fifth metatarsal.

**Table 3:** Skeletal dimensions as explained in the text.

Specimen	Femoral Length	Body Length	GAD/Body Length	Leg Length	Tail/Body Length
Carnegie	1.265	11.88	0.212	3.040	0.470*
Sue	1.312	12.29	0.225	3.300	0.512
Stan	1.278	11.78	0.211	3.068	0.594
MOR	1.280	11.60	0.157*	3.055	0.551
Jane	0.788	6.452	0.178	2.125	0.599

All units are in meters (except for ratios). GAD” is gleno-acetabular distance. The asterisk marks specimen measurements with unusual values relative to other specimens, discussed in the text.

These similarities and differences are reflected in the skeletal lengths and proportions: all four large tyrannosaurs had femur lengths around 1.3 m, body lengths 11–12 m and limb lengths 3–3.3 m, with Jane's lengths about 50–65% of these. The MOR specimen has an anomalously short body or torso (GAD), about 70% the length of the other three adults, whereas the fragmentary Carnegie specimen's extensive reconstruction is evident in its short tail length (<6 m and <50% of body length; all other specimens are closer to 7 m and >50% of body length). Again, the Sue specimen tends to have the greatest lengths of the four adult specimens and the other three are markedly similar in most dimensions. However Sue's tail is reconstructed beyond the 27^th^ caudal and could be artificially foreshortened as a result (cf. its tail length vs. that of Stan). Yet an alternative explanation, based on the number of actually preserved caudals (none in the CM 9380, 15 in MOR 555, 31 in BHI 3033, 36 in FMNH PR 2081), is that the seemingly foreshortened tail in the Sue specimen is actually more representative of the actual tail length, whereas other reconstructions have overestimated tail lengths. More complete *Tyrannosaurus* discoveries would resolve this issue more conclusively (see also discussion in [16]).

Overall, the best-preserved larger skeletons indicate that the torso elongated (by about 3% GAD/body length) [66] whereas the tail did not change length (remaining ∼60% body length) during ontogeny in *Tyrannosaurus rex*. Furthermore, unsurprisingly our skeletal data match Currie's ([17]; also [37]) finding that *T. rex* limbs experienced relative ontogenetic shortening (from 33% to 26% limb length/total length).

### Body and limb segment dimensions

Results for our five models are shown, segment by segment, in Table 4 for body (head to tail) and Table 5 for limb (pectoral limb and individual pelvic limb segments). Here we focus only on the “minimal” models, for brevity, but maximal model results are also included in Tables 4 and 5. Figures 2, 3, 4 show the resulting models.

**Table 4:** Body segment masses for the five tyrannosaur specimens.

Specimen	Segment	Model	Mass (kg)	Mass/Body Mass	Max/Min
Carnegie	Head	all	390	0.053	
Sue	Head	all	392	0.041	
Stan	Head	all	383	0.065	
MOR	Head	all	640	0.111	
Jane	Head	all	37	0.058	
Carnegie	Neck	min	365	0.049	
Sue	Neck	min	504	0.053	
Stan	Neck	min	190	0.032	
MOR	Neck	min	421	0.073	
Jane	Neck	min	30	0.046	
Carnegie	Neck	max	675	0.046	1.85
Sue	Neck	max	737	0.040	1.46
Stan	Neck	max	519	0.048	2.74
MOR	Neck	max	1005	0.093	2.39
Jane	Neck	max	75	0.058	2.53
Carnegie	Body	min	4271	0.578	
Sue	Body	min	5560	0.585	
Stan	Body	min	3214	0.542	
MOR	Body	min	2039	0.353	
Jane	Body	min	327	0.512	
Carnegie	Body	max	8606	0.591	2.01
Sue	Body	max	10011	0.541	1.80
Stan	Body	max	5269	0.486	1.64
MOR	Body	max	3597	0.334	1.76
Jane	Body	max	542	0.420	1.66
Carnegie	Tail	min	858	0.116	
Sue	Tail	min	1171	0.123	
Stan	Tail	min	587	0.099	
MOR	Tail	min	1196	0.207	
Jane	Tail	min	74	0.115	
Carnegie	Tail	max	2383	0.164	2.78
Sue	Tail	max	3477	0.188	2.97
Stan	Tail	max	1290	0.119	2.20
MOR	Tail	max	2375	0.221	1.99
Jane	Tail	max	168	0.130	2.28

Results for our minimal (“min”) and maximal (“max”) models are shown. Also displayed are segment percentages of body mass (“Mass/body mass”; for minimal or maximal models respectively; for head segment data are shown relative to minimal models) and the ratio of segment masses for maximal vs. minimal models (“Max/Min”).

**Table 5:** Limb segment masses for the five tyrannosaur specimens.

Specimen	Segment	Model	Mass (kg)	Mass/body mass	Max/Min
Carnegie	Forelimb	all	13	0.002	
Sue	Forelimb	all	11	0.001	
Stan	Forelimb	all	21	0.004	
MOR	Forelimb	all	18	0.003	
Jane	Forelimb	all	18	0.028	
Carnegie	Thigh	min	935	0.126	
Sue	Thigh	min	1287	0.135	
Stan	Thigh	min	1026	0.173	
MOR	Thigh	min	1008	0.174	
Jane	Thigh	min	99	0.155	
Carnegie	Thigh	max	1452	0.100	1.55
Sue	Thigh	max	2676	0.145	2.08
Stan	Thigh	max	2286	0.211	2.23
MOR	Thigh	max	2197	0.204	2.18
Jane	Thigh	max	277	0.215	2.80
Carnegie	Shank	min	410	0.055	
Sue	Shank	min	407	0.043	
Stan	Shank	min	342	0.058	
MOR	Shank	min	326	0.056	
Jane	Shank	min	38	0.059	
Carnegie	Shank	max	835	0.057	2.04
Sue	Shank	max	829	0.045	2.04
Stan	Shank	max	766	0.071	2.24
MOR	Shank	max	714	0.066	2.19
Jane	Shank	max	110	0.087	2.92
Carnegie	Foot	min	153	0.021	
Sue	Foot	min	123	0.013	
Stan	Foot	min	171	0.029	
MOR	Foot	min	129	0.022	
Jane	Foot	min	17	0.026	
Carnegie	Foot	max	209	0.014	1.37
Sue	Foot	max	196	0.011	1.59
Stan	Foot	max	300	0.028	1.75
MOR	Foot	max	222	0.021	1.72
Jane	Foot	max	41	0.032	2.48

Format as in Table 4. The “foot” segment represents the distal tarsals (when present), metatarsus and all preserved digits.

The heads of all four adult tyrannosaurs constituted 4.1–11.1% of the total (minimal) body mass, with the Jane specimen falling into the middle of that range (5.8%). The smaller values are for models made by VA+JM whereas the larger values come from KB's models and thus there may be some investigator bias in these models (see Discussion). Furthermore the MOR model by KB has an anomalously large head which is an artefact of the lower scan quality. The lower resolution of the raw scans results in a poor quality triangular mesh in the skeletal model of the MOR specimen [30], giving the digital skull bones an irregular topology. This error artificially inflates the mediolateral dimensions of the skull, creating both a larger external outline and smaller internal chambers, hence lowering the size zero-density respiratory structures. This has led to a substantially greater mass estimate—e.g., the density of the MOR reconstruction is 985 kg m^−3^ vs. 870 kg m^−3^ for Stan and 850 kg m^−3^ for Jane (cf. 791 and 807 kg m^−3^ for the Sue and Carnegie specimens by VA+JM). The head boundaries for Sue were derived from a CT scan of the mounted reconstructed skull, which attempts to correct for crushing and warping in the original. Although we acknowledge the potential for such reconstruction to affect overall volume, the ratio of head to body mass in Sue is roughly comparable to those of our Carnegie and Jane (and to a lesser degree, Stan) models.

Neck segment masses also varied widely, from 3.2–7.3% of body mass; again with Jane lying in the middle of this range (4.6%). The largest values are for the Sue and MOR specimens. The much larger value for the MOR neck segment compared to Stan and Carnegie again relates to their drastically different skeletal proportions, which determine the main body:neck segment lengths in our models. This difference (i.e., a relatively long neck, but shorter main body segment in MOR) is reflected in the relative masses of the main body and neck segments for MOR and Stan in the study of Bates et al. [30]. The caudal limit of the main body segment was defined by the caudal tip of the ischium, and the cranial limit by the cranial margin of the pectoral girdle (i.e., coracoids). The boundaries of the neck segment were defined by the latter boundary and the back of the skull. Thus any caudal shift of the pectoral girdle's boundary would increase the neck segment's volume. Therefore differences in mounted orientations of skeletal elements as well as missing/reconstructed elements, on top of real individual variation, contribute to these wide discrepancies.

The body (i.e., torso; base of neck to caudal end of sacrum) segment masses were remarkably similar for most specimens in terms of relative sizes, ranging from 54.2–58.5% body mass for the Carnegie, Sue and Stan specimens. Jane's body segment mass estimate was below this range (51.2%). However, clearly relating to its implausibly short torso (see above), the MOR specimen had a very small relative torso mass of 35.3% body mass. Almost 10% of the body mass that should have been apportioned to this segment instead ended up in the head and neck, plus more in the tail (below). We discuss this more later. Finally, even though they agreed on and used the same methods VA+JM and KB scaled their body segments differently: the maximal models were ∼1.9 times larger for the former but only ∼1.7 times larger for the latter (see Discussion).

The entire tail segment volume also showed wide variation corresponding to that observed for skeletal tail lengths (Table 3); 10–21% body mass, with Jane at 11.5% body mass. The models by VA+JM had similar relative masses (11.6–12.3%) and were scaled similarly for the maximal models (2.8–3 times more massive), whereas the models by KB had relatively smaller (9.9%; Stan) and larger (20.7%; MOR) masses and were scaled to maximal sizes by 2–2.2 times (2.3 times for Jane). Again the MOR estimates seem to have been skewed by different skeleton dimensions in particular segments, for the same reasons noted above.

Our analysis of main (axial) body segments raised the question: Do these apparent errors and biases persist in the limb segments? Pectoral limb masses of the adult specimens ranged from 0.1–0.4% body mass which we anticipated due to great subjectivity in assigning masses to the proximal forelimb vs. torso. Jane's forelimb mass was strikingly larger at 2.8% body mass, which did not seem to be an artefact of our methodology and fits well with ontogenetic changes discussed in the Introduction.

Pelvic limb masses, however, tended to be proportionately quite similar among the four adult *Tyrannosaurus* models. The thigh segment was the main exception – it ranged from 12.6%–17.4% body mass (with larger relative values for KB's models). Jane (with the relatively shorter femur; Table 2) had a relatively smaller thigh mass than KB's other models at 15.5%. These differences, and wide variation in scaling the thigh segment to maximal dimensions, are likely mainly caused by subjective investigator biases but also by large differences in how the torso was assembled in the various mounts, which affects overall mass and its proportional distribution. The shank mass estimates fell within a relatively narrow range of 4.3–5.9% body mass. Jane (with the relatively longest “tibiotarsus”; Table 2) was at the upper end of this range, again as expected. Finally, the foot (“tarsometatarsus” plus digits) segment masses varied from 1.3%–2.9% body mass, with the Sue specimen standing out as having a relatively small total segment. This was probably caused by differing subjective reconstructions including the inflated size of the torso, but also slightly by the absence of digit 1 and metatarsal 5 in the Sue -- and Carnegie-- specimens.

There were several obvious patterns of differences between our five models, which is most clearly summarized by considering the total masses dedicated to limb vs. body segments in the models by VA+JM and KB. Investigators VA+JM tended to produce models having 17.3–20.4% body mass in all four limbs, whereas KB's two adult *Tyrannosaurus* models did not overlap with this range (in minimal or maximal models) at 25.6–31.3% body mass, and his Jane model was larger (26.8–36.2% body mass). Conversely, the main body segments were relatively larger when constructed by VA+JM vs. KB. We consider these and other errors or biases in the Discussion.

### Body masses and COM positions

Table 6 shows our results for our five whole-body models. For the four adult *Tyrannosaurus* models we obtained a wide range of body masses and COM positions because our methodology purposefully produced wide “error bars.” We expanded our range of models to extremes that we qualitatively consider to be too skinny, too fat, or too disproportionate. We did this with the expectation that the actual (but effectively unknowable) body mass and COMs would fall within this range. Body mass (for minimal and maximal models; here rounded to nearest 100 kg) ranged from ∼5800–10800 kg for the comparably-sized Stan and MOR models made by KB, whereas the Carnegie and Sue models made by VA+JM produced proportionately larger masses: ∼7400–14600 kg for the former and ∼9500–18500 for the latter. For the Jane specimen we obtained masses of 639–1269 kg. Despite the occasional differences in individual body segment dimensions noted above, our maximal models were consistently around 1.9 times as massive as our minimal models.

**Table 6:** Body mass (all segments) and center of mass (COM) values shown for the five tyrannosaur specimens.

Specimen	Min mass (kg)	COMx (m)	COMy (m)	Max mass (kg)	COMx (m)	COMy (m)	Average mass (kg)	Mass range
Carnegie	7394	0.549	−0.332	14564	0.376	−0.347	9081	1.97
Sue	9502	0.801	−0.355	18489	0.572	−0.356	13996	1.95
Stan	5934	0.524	−0.495	10837	0.504	−0.521	8385	1.83
MOR	5777	0.572	−0.337	10768	0.386	−0.387	8272	1.86
Jane	639	0.276	−0.318	1269	0.242	−0.307	954	1.98

Shown are COM positions (relative to the right hip joint; cranial  =  +x; ventral  =  −y) for our minimal (“Min”) and maximal (“Max”) models, with averages of minimal and maximal model body masses as well as the ratio of maximal to minimal masses (“Mass range”).

Estimated COM position varied relatively more among the four adult *Tyrannosaurus* models than among different iterations of individual specimens. However, the similar-sized Carnegie, Stan and MOR specimens, despite apparent investigator bias resulting in different body mass estimates, had roughly comparable cranial positions of the COM (∼0.55 m from hip) for the minimal models. The pronounced difference of these models' COM cranial positions from the Sue model (0.80 m) can be partly explained by the greater size of the FMNH skeleton -- with contributions from errors such as the artefactually wide, tall ribcage (see above and Discussion). Likewise, the cause of the ∼50% more ventral COM position in the Stan model (0.495 m vs. ∼0.34 m below the hips in the other three large models) is unclear but likely a result of modelling errors.

Absolute COM positions are shown in Table 6, but in Table 7 we focus on relative COM positions, using a variety of normalizing metrics (following [31]). Here we focus on the results for femur length as a normalizing factor because a femur is well preserved for all specimens whereas the other metrics are more likely to have been influenced by skeletal preservation and mounting biases, although they are all presented in Table 7. Moderate variation in COM position was found along the y (dorsoventral) axis between the different iterations of each specimen, but the dorsoventral COM position varied more widely between specimens. Expressed as % femur length ventral to the hip, the COM was estimated to be 22–35% for the Carnegie and MOR specimens, a surprisingly conservative but otherwise similar 26–28% for Sue, 38–50% for Stan, and 35–54% for Jane. As expected from previous studies [28], [31], we found greater variation in craniocaudal (x-axis) COM position for each of our specimens. Again using femur length to normalize the results, the COM distance from the hip joint ranged from −1.9% (i.e., behind the hip; this highly implausible result was only found for the Carnegie specimen) to 88.8% (in Sue specimen) femur length in “Most Cranial” and “Most Caudal” extreme models. These were intentionally designed (see Methods) to maximize the possible range of COM positions given a set value for morphological variability (here, ±20% radial dimensions for body segments), rather than to satisfy the valid but hard to quantify criteria of appearing ‘natural’ (subjectively plausible to an observing anatomist). Hence they should not be assumed to be equally as plausible as less circumspect, ‘natural looking’ models. Instead, the range of ∼30–45% femur length for most minimal and maximal (also most dorsal and ventral) models; albeit with Sue at 44–61%; would cover our qualitative assessment of the most plausible COM range for adult *Tyrannosaurus*. Jane's COM estimates (30–35% femur length) fall at the lower boundary of this range, but as these ranges overlap appreciably it is not conclusive whether the COM experienced a craniad ontogenetic shift (0–15% femur length).

**Table 7:** Whole body masses and center of mass (COM) positions for the five tyrannosaur specimens.

Z	Body mass (kg)	COM distance from hip (m)		COM (% Femoral length)		COM ( % Body length)		COM (% GAD)		COM (% Leg length)	
		X	Y	X	Y	X	Y	X	Y	X	Y
***Carnegie***											
Most Cranial	11880	0.827	−0.275	65.4	−21.7	7.0	−2.3	33.2	−11.1	27.2	−9.0
Most Caudal	10390	−0.024	−0.395	−1.90	−31.2	−0.20	−3.3	−1.00	−15.9	−0.8	−13.0
Most Dorsal	13405	0.428	−0.300	33.8	−23.7	3.6	−2.5	17.2	−12.1	14.1	−9.9
Most Ventral	8888	0.447	−0.388	35.3	−30.7	3.8	−3.3	18.0	−15.6	14.7	−12.8
Maximal	14564	0.376	−0.347	29.7	−27.4	3.2	−2.9	15.1	−13.9	12.4	−11.4
Minimal	7394	0.549	−0.332	43.4	−26.2	4.6	−2.8	22.1	−13.3	18.1	−10.9
***Sue***											
Most Cranial	14300	1.160	−0.361	91.2	−25.5	9.7	−2.7	43.2	−12.1	36.3	−10.2
Most Caudal	13691	0.116	−0.350	13.6	−24.7	1.4	−2.6	6.40	−11.7	5.4	−9.8
Most Dorsal	16605	0.641	−0.358	51.5	−25.4	5.5	−2.7	24.4	−12.0	20.5	−10.1
Most Ventral	11386	0.662	−0.352	55.5	−24.8	5.9	−2.6	26.3	−11.7	22.1	−9.8
Maximal	18489	0.572	−0.356	46.0	−24.9	4.9	−2.7	21.8	−11.8	18.3	−9.9
Minimal	9502	0.801	−0.355	66.8	−25.5	7.1	−2.7	31.7	−12.1	26.6	−10.2
***Stan***											
Most Cranial	8319	0.869	−0.511	68.0	−40.0	7.4	−4.3	34.9	−20.6	28.3	−16.7
Most Caudal	8450	0.154	−0.602	12.1	−47.1	1.3	−5.1	6.20	−24.2	5.0	−19.6
Most Dorsal	9023	0.622	−0.488	48.6	−38.2	5.3	−4.1	25.0	−19.6	20.3	−15.9
Most Ventral	7746	0.378	−0.637	29.5	−49.9	3.2	−5.4	15.2	−25.6	12.3	−20.8
Maximal	10837	0.504	−0.521	39.4	−40.8	4.3	−4.4	20.3	−20.9	16.4	−17.0
Minimal	5934	0.524	−0.495	41.0	−38.7	4.4	−4.2	21.1	−19.9	17.1	−16.1
***MOR***											
Most Cranial	7918	0.881	−0.279	68.8	−21.8	7.6	−2.4	48.3	−15.3	28.8	−9.1
Most Caudal	8626	0.056	−0.453	4.40	−35.4	0.5	−3.9	3.1	−24.8	1.8	−14.8
Most Dorsal	9098	0.511	−0.321	39.9	−25.1	4.4	−2.8	28.0	−17.6	16.7	−10.5
Most Ventral	7447	0.378	−0.428	29.5	−33.5	3.3	−3.7	20.7	−23.5	12.4	−14.0
Maximal	10768	0.386	−0.387	30.2	−30.2	3.3	−3.3	21.2	−21.2	12.6	−12.7
Minimal	5777	0.572	−0.337	44.7	−26.3	4.9	−2.9	31.4	−18.5	18.7	−11.0
***Jane***											
Most Cranial	975	0.428	−0.286	54.4	−36.3	6.6	−4.4	37.3	−24.9	20.2	−13.5
Most Caudal	1009	0.080	−0.397	10.2	−50.4	1.2	−6.2	7.0	−34.6	3.8	−18.7
Most Dorsal	1069	0.298	−0.272	37.8	−34.5	4.6	−4.2	26.0	−23.7	14.0	−12.8
Most Ventral	915	0.192	−0.423	24.4	−53.7	3.0	−6.6	16.8	−36.9	9.1	−19.9
Maximal	1269	0.240	−0.294	30.5	−37.3	3.7	−4.6	20.9	−25.6	11.3	−13.8
Minimal	639	0.276	−0.318	35.0	−40.3	4.3	−4.9	24.0	−27.7	13.0	−15.0

Body mass, absolute COM position, and COM position are shown; the latter is normalized by four alternative metrics: femur length, body length, gleno-acetabular distance (GAD), and limb length (data from Table 3). The six models represented for each specimen are four variants maximizing the cranial, caudal, dorsal and ventral COM extreme positions, and the minimal and maximal mass models.

### Reconstructed M. caudofemoralis longus (CFL) muscle masses

**Figure 5:**
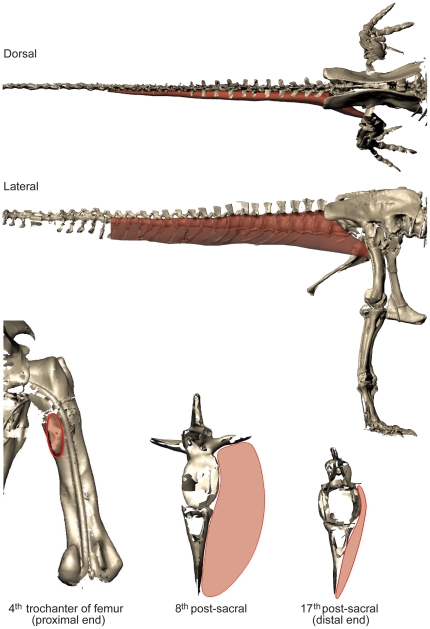
Muscle mass reconstruction method for M. caudofemoralis longus (see Methods); Carnegie specimen depicted. Dorsal and right lateral views are shown on top, and in the bottom row are caudal views of the right femur and then caudal vertebrae (8^th^ and 17^th^). Red shaded volumes are the M. caudofemoralis longus reconstruction. Note a small space for M. caudofemoralis brevis (not reconstructed) is left around the ilium/sacrum and lateral to the CFL insertion.

Our models of four adult *Tyrannosaurus* had remarkably consistent CFL muscle mass estimates (Figure 5; Table 8) of 162–189 kg for minimal models (the smaller value surely caused by the smaller tail segment of MOR). The relative masses ranged from 1.95–3.9% body mass for all the different size (Min and Max) models. This contrasts with the potentially slightly larger CFL muscle mass in Jane (at 3.4–3.8% body mass).

**Table 8:** M. caudofemoralis longus (CFL) muscle masses for the five tyrannosaur specimens.

Specimen	CFL mass (kg)	% Body Min	CFL mass Max	% Body Max
Carnegie	187	2.53	520	3.57
Sue	189	1.99	561	3.03
Stan	192	3.24	22	3.90
MOR	162	2.80	322	2.99
Jane	34.0	5.32	77.4	6.10

Initial (corresponding to Minimal model; “Min”) and maximal (“Max”; multiplied by the ratio of Maximal/Minimal tail segment masses in Table 4) masses are shown; also normalized as a percentage of body mass.

Our validation test for the extant *Crocodylus johnstoni* specimen indicated reasonable accuracy for our methodology, concurring with a similar reconstruction technique [19]. The estimated muscle mass was 0.311 kg and the measured mass was 0.316 kg, although the assumed density (1000 kgm^−3^) was lower than the likely actual density (∼1060 kgm^−3^); a more comparable estimate would be 0.330 kg (4.4% overestimate of volume or mass vs. actual). This corresponds to a CFL mass of 1.6% body mass (per hindlimb) for the adult crocodile; identical to values in adult *Alligator* [54].

### Muscle masses from segment minus bone volumes

By subtracting bone volumes from thigh and shank segment volumes, then adjusting these volumes to estimate extensor muscle masses, we aimed to derive more accurate muscle mass estimates for *Tyrannosaurus*. Generally, the lower end range of our estimates (Table 9) compares reasonably well with others' [28] (total extensor masses per limb of 12–14% body mass). Total extensor muscle masses estimated for each of the three major joints of one pelvic limb varied in similar ways for each of our models (Table 9). The hip joint extensor muscle masses, including our CFL estimates, ranged from 8.8–15.1% body mass for the adult tyrannosaurs and 11.6–15.9% for the Jane specimen. The knee extensor masses ranged from 3.3–7.1% body mass in adults vs. 4.9–7.3% for Jane (squarely overlapping the results for other models made by KB). Finally, the ankle extensor masses ranged from 1.9–3.2% body mass in adults vs. 2.5–3.9% for Jane. Thus the total extensor muscle masses for one hindlimb were about 14–25% body mass for adults vs. 19–27% body mass for the Jane specimen. These estimates naturally reflected similarities and differences noted above for CFL mass and segment mass ranges for the adults vs. the juvenile. They are heavily dominated by the hip/thigh musculature including the CFL (proportion of total limb extensor mass roughly 60% hip vs. 40% knee and ankle). Our estimates do not consider the small (∼6%) difference between the density of our models' homogeneous flesh (1000 kg m^−3^ not including air cavities, which are negligible for the limbs and tail) and the density of vertebrate muscle.

**Table 9:** Estimates of extensor muscle masses (total acting about each joint in one hindlimb) for tyrannosaur specimens.

	Extensor mass as % body mass per joint				
Joint	Carnegie	Sue	Stan	MOR	Jane
Hip min	9.05	9.10	12.3	11.9	13.2
Hip max	8.79	10.7	15.1	13.9	17.6
Knee min	4.10	4.40	5.68	5.76	4.94
Knee max	3.29	4.80	7.07	6.84	7.27
Ankle min	2.33	1.90	2.52	2.42	2.46
Ankle max	2.55	2.00	3.22	2.99	3.93
**TOTAL MIN**	15.5	15.4	20.5	20.1	20.6
**TOTAL MAX**	14.6	17.6	25.4	23.7	28.8

As described in the methods we estimated muscle volumes from limb segment volumes (minus bone volumes, which were small fractions (∼5%) of the segment volumes) as in Hutchinson et al. (2007). We adjusted those non-bony segment volumes by multiplying them by the percentages of segment mass observed in extant Sauria (based on dissection data from Hutchinson, 2004a,b) that are dedicated to extensor muscles. The hip extensor estimate was 54% of thigh mass (plus CFL mass from Table 8), the knee extensor estimate was 34% of thigh mass, and the ankle extensor estimate was 47% of shank mass.

### Growth

**Figure 6:**
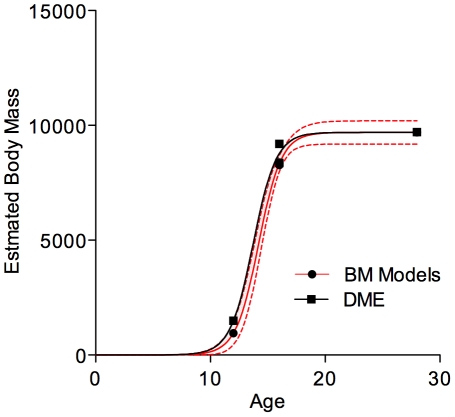
Adjusted growth curve (mass in kg as a function of age in years) for *Tyrannosaurus rex*. Filled circles represent our mass estimates derived from digital modelling; squares represent data points generated using Developmental Mass Extrapolation [42]. The red line represents the best fit curve for modelled data; the black one is for the DME estimate. Stippled lines represent the 95% confidence intervals for the model-based estimates. The MOR specimen is treated as being 16 years old in this plot (see text).

Given the overall larger specimen masses derived from our modelling of scans of mounted specimens, it comes as no surprise that the adjusted growth curve for *Tyrannosaurus rex* finds significantly higher growth rates during the exponential phase (Fig. 6), than previously reported [39]. Using the model based mass estimates, maximum growth rates for *T. rex* during the exponential stage are 1790 kg/year-- a more than twofold increase from the reported value [39]. Comparisons between model-based and DME mass estimates are relatively favourable. The DME-derived estimates fall within the 95% confidence limit when the MOR specimen is assumed to be 14 years of age, though not if it is considered to be 16 years old (Fig. 6). Under the latter scenario, its mass and age are almost identical to that of Stan, essentially removing any scatter in a plot with so few data points. Both the AICc and F-tests overwhelmingly favour a single model (i.e., one growth curve) to fit both data series.

## Discussion

### What were the body segment dimensions of an adult *Tyrannosaurus rex* and how might they have changed during ontogeny?

This was the primary question of our study. Although our results are complicated by specimen variation, incomplete preservation, mounting errors and investigator biases (discussed below), there are some general patterns that we infer to be indicative of potential ontogenetic changes in the morphology of *T. rex*. It is not clear how much (if at all) head, neck or tail mass changed, but the forelimbs clearly became proportionately lighter from juveniles to adults (by as much as a factor of 10), the torso (despite uncertainties about gastral basket shape) became longer and thus heavier (by perhaps 5% of body mass) and the limbs became relatively lighter overall (but presumably heavier for the longer thigh/femur). These trends were suggested by skeletal data (including our Table 3) but reinforced by our “fleshed-out” models (Tables 4,5), across an order of magnitude change in body mass (Table 6). Resulting whole-body density values for all five minimal models overlap those of previous studies [28], [30]: ∼840–890 kg m^−3^; vs. less plausible values of ∼970 kg m^−3^ for the maximal models.

These changes of proportional masses may have altered the body's COM position. Discounting the anomalous results for the Stan specimen (noted above), our results indicate that a dorsal shift of the COM position in tyrannosaur ontogeny (by <33% femur length; Table 7) is the most plausible conclusion. The COM also may have shifted slightly cranially; possibly by as much as 15% femur length; although the latter shift is more ambiguous and deserves closer examination in the future. However it would be interesting whatever result was supported-- the two extant archosaurs examined to date seem to show a craniodorsal shift of COM position during ontogeny [31] so *T. rex* might be unusual if it did not experience such a cranial shift. Contrarily, if there was a cranial shift of COM position it could have altered locomotor abilities, as discussed below.

### Sources of error in body segment reconstructions of tyrannosaurs

The error in any reconstructions of extinct organisms must be addressed as part of interpretations of their biology that rely on those reconstructions, so we consider this problem in relation to the primary aim of our study (above). We discuss two major sources of error here; neither was discussed in any detail by our preceding studies [28], [30]–[31], [35]. First, while our approach offers more precision in terms of gauging individual and ontogenetic variation than many previous methods of mass estimation (see below), it certainly is sensitive to how specimens were restored and reconstructed. Second, investigator biases in digitally reconstructing our specimens to create volumetric models are also evident. Other sources of error such as whole body density (similar for all specimens here although varying for some segments such as the head; see above) and body segment orientations (generally standardized here) were previously addressed [31], so we avoid repetition here.

**Figure 7:**
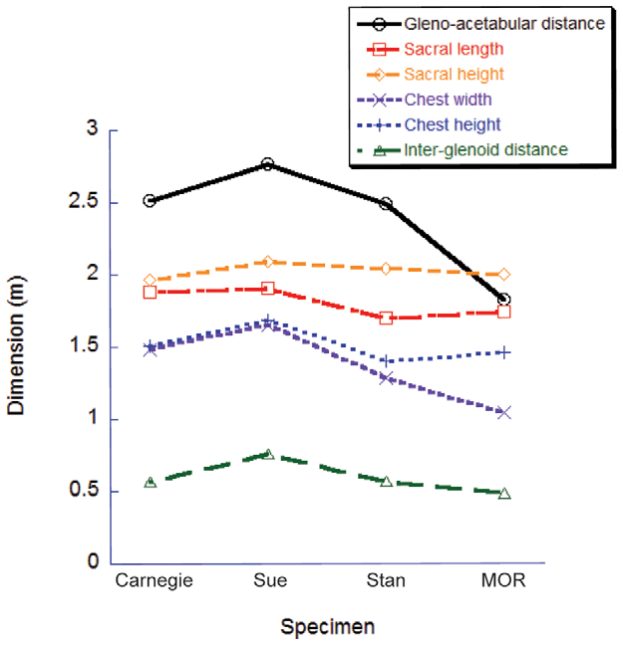
Comparison of torso/body dimensions for the four large *Tyrannosaurus rex* specimens. Linear measurements from our digital models show how the gleno-acetabular distance (GAD) is anomalously short in the MOR specimen and the chest is anomalously wider in the Sue and Carnegie specimens. Otherwise all four specimens compare fairly well, considering that the Sue specimen is known to be somewhat larger.

Although relatively little difference exists between the four adult specimens in most linear dimensions, they do differ considerably in the shape and volume of their torso, due to conflated biases of incomplete preservation (and thus varying reconstructions), distortion (of preserved or reconstructed elements) and different mounting techniques (articulations of preserved or reconstructed skeletal elements). This is particularly germane to Sue, and to a lesser degree the Carnegie specimen. Both are mounted with a proportionately longer, wider, and taller torso than the others (Fig. 7). This barrel-chested reconstruction in Sue is an artefact of the dorsal displacement of the transverse processes on the trunk vertebrae, which forced a dorsal displacement of the tubercular articulations and a lateral expansion of the rib cage as a whole. Better understanding of the anatomy of the partial gastral basket (a problem for all our specimens) and potential span of the probable, but pathological, furcula [73] may help constrain the span of the front end of the rib cage and correct the effects of the inflated chest dimensions. Because the torso is by far the largest body segment, overall body mass and the mass distribution between segments should be strongly affected by changes in its dimensions. However, one study of *Allosaurus* [35] found that this effect was smaller than one might expect, largely because the zero-density lungs and air sacs were expanded with the ribcage in their models. Indeed, the error caused by the large ribcage in our study may have more to do with our coarse distribution of octagonal hoops used to loft flesh around skeletal landmarks (see Methods and Figure 1), but a much broader sensitivity analysis and validation study would be needed to test this.

We also commented above on other problems with our various specimens, such as the short torso and large head, neck, and tail in the MOR specimen (e.g., >100% difference of relative masses of head and neck segments in MOR vs. Stan specimens). These problems of incomplete preservation and unstandardized mounting techniques plague all anatomical reconstructions and thus body mass estimates of tyrannosaurs. They thereby limit our understanding of intraspecific variation, such as the likely range of body segment dimensions in adult *Tyrannosaurus* or ontogenetic changes of morphology [5], [16], [66]. The best-preserved specimen for our study's purposes appears to be the Stan specimen but even it displays some anomalous results; e.g., its ventral COM position.

Subjectivity is unavoidable in our reconstructions, so different investigators might produce quite different models. Investigators VA+JM and KB each reconstructed two adult tyrannosaur specimens. The former investigators tended to produce more “fleshy” reconstructions (except for the head segment), partly caused by specimens with more barrel-chested torsos (see [26], [30], [35]), but also influenced by subjective judgements about segment dimensions that are difficult to estimate from skeletal dimensions. In some cases (especially the tail and thigh segments; less problematically the “foot” segment), the difference between adult tyrannosaur models made by different investigators was considerable and could not be ascribed to the above preservational/mounting biases. Furthermore, although our two investigator teams agreed on similar methods in advance there were clearly reproduced differences in how they executed them. For example, KB tended to use more “hoops” than VA+JM, producing more skeleton-hugging body outlines, and had a pronounced narrowed “waist” between the pelvis/sacrum/limbs and the chest (Figs. 2, 3, 4), which is likely a partial explanation for KB's tendency to produce smaller main body segments and differently proportioned leg segments (including min/max scaling differences; Tables 4,5) from VA+JM.

Importantly, our minimal models are much more plausible reconstructions than our maximal models, especially for the Carnegie and Sue specimens, considering the realistic density (see above; similar to extant archosaurs'; [31]), less ridiculously rotund appearance (Figs. 2, 3, 4) and smaller masses (closer to previous studies' estimates) of the minimal models (Table 6). We certainly do not contend that our maximal models are results that should be assumed to be equally as valid as our minimal models. Rather, they form extreme upper end values on a spectrum of “fleshiness.” The actual mass of individual *Tyrannosaurus* specimens should have fallen well below these extremes and much closer to our minimal models', but how much below may never be known. Likewise, as noted above our maximal cranial, caudal, dorsal and ventral models (Table 7) are not as plausible as our minimal models, but rather form extreme boundaries that fairly fully explore the impact of reconstructions on mass, mass distribution and COM position. Regardless of errors in the reconstruction, even the large mass estimate (9502 kg) of the minimal model of the Sue specimen remains a plausible value. Yet considering those errors, the actual mass might have been smaller, whereas the minimal reconstructions by KB could be viewed as lower-end estimates.

At present there is little that can be done about such subjectivity except to be aware of it and explicitly acknowledge it, but there is no reason that improvements could not be made in the future. One potential corrective method is that there could be consistent relationships between bone and “fleshed-out” outlines of skeletons in extant taxa that could be quantified and applied to extinct taxa. Allen et al. [31] proposed one fairly simple approach for saurian tails that led to more realistic reconstructions, even though our two teams of investigators still produced somewhat different tail dimensions due to some lingering subjectivity in that method.

### What body mass estimate methods are most reliable?

Our methods again raise this question that has been recurring in recent studies. Even with consideration afforded to artefacts of reconstruction and investigator biases in fleshing out skeletons (above), it is clear that even minimal body mass values for *Tyrannosaurus rex* estimated directly from skeletons tend to be larger than published values extrapolated from scale models or limb bone dimensions [9]–[12], [32], [36], [74]. This suggests that researchers have tended to favour ‘skinnier’ reconstructions in the production of scale models, with scaling perhaps magnifying the effects of such subjective choices. Our results (focusing on our minimal models which are more plausible as noted above and below) overlap better with those that employ graphic integrations [26], [75], though they are overall still greater in mass. They compare well with those of other computer volumetric studies using similar methods [28], [30].

As others have noted (see discussion in Hutchinson et al. [28]:p.674), 3D digital reconstruction-based estimates give more plausible results than scaling equations because they are specimen-specific and take into account the unique whole-body morphology and density (as well as explicit sensitivity analysis thereof) rather than assume a very tight relationship between one skeletal dimension and body mass for many species. They thus sidestep the “everyanimal” problem adduced by Pagel [76]. Furthermore the mass estimate equations such as Anderson et al. [32] have other errors and assumptions that leave their merits highly questionable. For example, a classic study [32] adjusted its mass estimates for bipeds using scale models of bipedal dinosaurs whose methodological accuracy is uncertain at best; dubious at worst. The statistical inaccuracies of the same study have also been a subject of recent debate [33], [77]–[78]. Of course, there are no error- or assumption-free methods for estimating dinosaur body dimensions. Although in some cases our results are biased by excessively bulky reconstructions, such as in the case of the Sue specimen, our mass estimates for *T.rex* derived from actual skeletal data are markedly heavier than proposed by several commonly used equations. These equations have been employed in understanding growth and other life history parameters for this taxon, so next we consider how their usage has influenced estimates of tyrannosaur biology.

### Implications for growth rates

The second aim of our study was to revisit published *Tyrannosaurus rex* growth rates with consideration of the validity of the DME approach as well as the accuracy of body masses used in such approaches. Erickson et al. [39] noted that in spite of its impressive magnitude, their estimate of *T. rex* maximal growth rate was only between a third and half of the expected value for a non-avian dinosaur of that size, when compared to regressions relating peak growth rate to body mass. Our new value for peak growth rate (Fig. 6) would largely erase the reported difference between observed *T. rex* peak growth rates and the expected theoretical value. Because the latter relationship was calculated using mass extrapolations as a function of limb bone diameters [32] rather than modelling, however, we cannot be sure how much confirmation of our mass estimation this seemingly better-fitting result offers. Considering that the upper end of the published theoretical regression [38] was defined by quadrupedal sauropodomorphs, whose masses are likely underestimated by the Anderson et al. equation ([32];see above), our greater estimate is still in keeping with what is to be expected of growth patterns in non-avian dinosaurs.

One effect of finding a faster peak growth rate is that the age at which 50% of adult body mass is reached is shifted earlier into ontogeny. Compared with Erickson et al. [39] who found that *Tyrannosaurus rex* reached this rate around age 16, our results indicate that this threshold is passed between the ages of 12.9 and 14.2 years depending on how the age of the MOR specimen is set. The lower estimates seems unrealistic given the calculated mass of the Jane specimen, which is a 12 year old individual, but the upper bound appears reasonable if an animal of Jane's size is given two years to grow at or near the peak rate. Our results support the inference of an earlier attainment of somatic maturity (growth asymptotes) at around 16–17 years of age compared to the 18–19 years previously estimated from Sue's histology [39]. Given the paucity of data points, it is prudent to interpret the extrapolated growth curve in light of actual specimen data, which would slightly decrease peak growth rates and increase the age at which 50% of mass is reached. We did not implement such ‘biological’ corrections here, as our focus was on estimating the raw effects of change due to new mass estimates and also to compare our values with those estimated using DME. Likewise, we have not investigated the impact of our results on other inferences made based on these mass estimates and growth rates, such as the correlation of ageing rates with mortality [79]–[80].

With respect to the latter, our results, limited as they are, indicate that the DME method represents a robust first approximation for deriving masses for somatically immature ontogenetic stages of bipedal dinosaurs. Statistical evaluation of model fitting indicates that there is no significant difference between the constants for growth curves fitted to model-based data and to data generated by applying DME to the minimum body mass of Sue. The DME-generated results also fall within the 95% confidence interval for one of the possible age resolutions for the MOR specimen, though not the other, further bolstering our conclusion. Nevertheless, some caution is warranted as we were forced to use different estimates (i.e., minimum mass value) for Sue as opposed to the other specimens, for which we plotted average values. Although this fact was dictated by the exaggerated reconstruction of Sue's ribcage, we have no means to evaluate how much of an effect that would have. In our favour, however, is the observation that using the minimum value for Sue does result in a growth curve with an asymptote that reflects histological evidence (e.g., the external fundamental system) for a prolonged somatic stasis in Sue. Though more data are required to fully test the precision of DME for *T. rex* and other taxa, our results are generally encouraging.

### Did locomotor performance change during *Tyrannosaurus* ontogeny?

The third aim of our study was to test two related hypotheses about tyrannosaur ontogeny (Was there a relative decrease in hindlimb muscle mass? Did COM position shift cranially?), inspired by ontogenetic changes of relative segment dimensions that are indicated by gross skeletal shapes. Unfortunately, our reconstructed CFL masses and other hindlimb muscle mass data are somewhat ambiguous for juvenile vs. adult tyrannosaurs. Although the upper end of the range of muscle mass estimates for the Jane specimen tended to be relatively larger (as % body mass) than for the adult specimens (especially for the more rotund-bodied models of VA+JM), the ranges overlapped (CFL: ∼2–4% body mass in adults vs. 3–4% in Jane; all limb extensors: 14–25% body mass in adults vs. 19–27% in Jane). Thus, although there is some tentative support for the hypothesis that limb muscle mass decreased during ontogeny (by as much as double across a tenfold change of body mass), the null hypothesis of no difference is not excluded. Because the distal end of the tail is the most frequently missing part of the tail in our specimens, into which the CFL muscle would not have extended, the poor preservation of the tail (discussed above) is not a severe problem for our estimates of CFL muscle volume. Nonetheless, estimates of CFL masses in *Tyrannosaurus* deserve reconsideration as better specimens become available.

However our results cast doubt on the inference of Persons and Currie [19], who used one small *Gorgosaurus* and one *Tyrannosaurus* specimen with no sensitivity analysis of error in their estimates, to infer that CFL muscle relative masses increased across tyrannosaurid ontogeny. Their hypothesis is not conclusively falsified either, but is less supported than an ontogenetic increase or lack of change, for the following reasons (other issues with their study were raised elsewhere [81]) in addition to our larger dataset.

First, Persons and Currie [19] used smaller body mass estimates (3800–4500 kg) for *Tyrannosaurus* [81], which inflates the percentage of body mass that their reconstructed CFL muscle seems to represent. For example, if one assumes a body mass of 4500 kg for the Stan specimen then a 261 kg CFL muscle [19] makes up 5.8% body mass. If one instead assumes 7600 kg ([30]; in the middle range of this study's estimates from 5934–8385 kg) then that same 261 kg CFL muscle mass only makes up 3.4% of body mass, or 2.5% if this study's smaller estimate of 192 kg is used. Thus a factor of over 100% error can be introduced into relative muscle mass comparisons by error in body mass estimates.

Second, our CFL reconstruction method obtained generally smaller mass estimates for CFL (see example above and Table 8) than Persons and Currie [19] did, for several reasons. These include that we did not also reconstruct the smaller M. caudofemoralis brevis (Fig. 5), and our reconstruction was not constrained to semicircular shapes but rather involved smooth ellipses. Our minimal estimates have a closer match to values obtained with the simpler (25% tail base segment mass) approach of Hutchinson et al. ([28]; ∼141 kg) but are superior in being more anatomically realistic and reasonably well validated.

Likewise, the results of our COM position estimates for adult and juvenile *Tyrannosaurus* are not conclusive, but hint at the potential for a craniad (and dorsal) shift during ontogeny, as noted at the beginning of the Discussion above. If the latter was the case, then this would require greater active muscle volumes around the hip to stabilize the COM and could increase functional demands for muscles acting around other joints [21], [25], just as those muscle masses are potentially decreasing (as above). That would likely result in a steeper decrease of maximal locomotor performance (e.g., running speed) across tyrannosaur ontogeny.

Overall, however, the consequences of our muscle and COM estimates for the ontogenetic scaling of locomotion in tyrannosaurids remain ambiguous. Most animals maintain, or slightly decrease, absolute maximal locomotor performance during growth while reducing relative maximal performance (e.g., maximal ground reaction forces sustainable, or maximal Froude number; or minimal duty factor; attainable) [22], [49]–[55], [82]–[83]. Our results are very consistent with these general patterns, and are bolstered by the biomechanical analyses of Hutchinson [21] and the scaling of the limb skeleton [17], [37] across tyrannosaur ontogeny. There is however no strong evidence for whether absolute maximal performance decreased or not, but there is little reason to doubt that relative maximal performance declined. To better resolve this mystery we suggest at least the following are needed: (1) sufficiently large sample sizes of 3D models of tyrannosaurs to properly characterize ontogenetic scaling patterns (once sufficient specimens exist; see [17], [37] for initial skeletal studies); (2) more rigorous validation techniques and sensitivity analysis for estimating muscle and body dimensions (including statistical treatments of these linked to point #1); and (3) improved, well-validated biomechanical models of tyrannosaur locomotion across ontogeny that test the influences of these dimensional estimates on quantitative locomotor performance.

### Comparative analysis of limb muscle masses

Our method for estimating limb muscle masses refines previous estimates [19], [28] but is not without its own problems. Although our CFL muscle mass estimation method seems reasonably sound (∼4% error; comparable to 6% error by others [19]), our estimates of total limb extensor mass are admittedly crude. They assume that extensor muscles are a relatively constant fraction of segment mass (excluding bones; a novelty of our analysis) in the clade Sauria, especially within Theropoda. There is no conclusive evidence supporting this; it is merely a plausible and simple assumption. Our method also makes numerous assumptions (e.g., ignoring masses of integumentary, connective tissue, or circulatory tissues) that bias our results toward overestimates.

With the above caveats in mind, our tentative conclusions are still quite interesting. Table 10 shows a wide range of data for limb muscle and segment masses in extant amniotes for comparison with our *Tyrannosaurus* estimates. If the lower ends of our estimates are considered plausible (and even these may be overestimates), then *Tyrannosaurus* may have had limb extensor muscles that were proportionately as large as those of any extant animal including ratites. Those muscles may have even been proportionately twice as large as those (for all limbs) in large quadrupedal mammals such as giraffes, rhinoceroses, hippopotami and elephants. If the upper ends of our estimates are upheld by future studies, then *Tyrannosaurus* (and perhaps other non-avian theropods) may, in relative terms, have had the largest limb muscles of any known land animal. However, we urge caution to avoid excessively credulous attitudes toward these estimates for reasons noted above. Furthermore, as Hutchinson et al. [28] and other studies have noted, the notion that extinct theropods were more muscular than extant ratites strains plausibility, because the small heads and necks, short forelimbs, vestigial tails, extremely elongate pelvic limb bones and other features of ratites are a bauplan more amenable to maximizing the relative masses of pelvic limb muscles -- except the CFL.

**Table 10:** Muscle and limb masses in select amniotes.

Genus	Refs	M_body_ (kg)	Clade	Sampled Limb	M_extensor_ / M_body_ (%)	M_musc_ /M_body_ (%)	M_limb_ / M_body_ (%)
*Gecko*	[88]	0.057	Squamata	Pelvic	5.1	17	
*Basiliscus*	[67]	0.191	Squamata	Pelvic	6.9	19	26+
*Iguana*	[67]	4.04	Squamata	Pelvic	2.5	6	15+
*Alligator*	[54], [67]	10	Crocodylia	Pelvic	5.4	15	9.7+
*Coturnix*	[87]	0.095	Aves	Pelvic	5.5	11	
*Pica*	[89]	0.22	Aves	Pelvic	2.8	7	18
*Eudromia*	[67]	0.406	Aves	Pelvic	4.2	11	19
*Numida*	[90], [91]	1.47	Aves	Pelvic	9.1	23	27
*Gallus* (junglefowl)	[84]	1.94	Aves	Pelvic	6.6	16	
*Meleagris*	[67]	3.7	Aves	Pelvic	5.5	12	20
*Dromaius*	[67]	27.2	Aves	Pelvic	14.5*	28	41
*Struthio*	[67]	65.3	Aves	Pelvic	10.5	26	38
*Lepus*	[92], [93], [98]	3.45	Mammalia	Pect+Pelv	10.2	27	29
*Macropus* ^1^	[67], [86]	6.6	Mammalia	Pelvic	9.1	22	36
*Macropus* ^2^	[86]	10.5	Mammalia	Pelvic	9.6	22	
*Canis* (greyhound)	[94], [95]	31.6	Mammalia	Pect+Pelv	12.5	36	33
*Acinonyx*	[96], [97]	33.1	Mammalia	Pect+Pelv	11.2	32	40
*Pan*	[99], [100]	54.6	Mammalia	Pect+Pelv	11.6	32	40
*Homo*	[67]	71	Mammalia	Pelvic	9.5	9	39
*Equus*	[85], [101]–[103]	510	Mammalia	Pect+Pelv	10.0	28	30
*Giraffa*	JRH	684.5	Mammalia	Pect+Pelv	11.3	32	
*Hippopotamus*	JRH	1600	Mammalia	Pect+Pelv	7.3	21	
*Ceratotherium*	JRH	1755	Mammalia	Pect+Pelv	6.8	20	
*Elephas*	JRH	3550	Mammalia	Pect+Pelv	6.7	20	27.7
*Tyrannosaurus rex*	This Study	5934–18611	Tyrannosauridae	Pelvic	≤14.6–25.4	?	35–65
**Mean**					8.1	20	29
**SD**					3.1	8.5	9.6

“Refs” lists the literature source for the data; “JRH” indicates specimens from author JRH's personal collection, from single musculoskeletally sound zoo-sourced adult specimens, and are otherwise unpublished data. “**Sampled** **Limb**” indicates which limbs data are shown for; in particular note that only pelvic limb data are available for the first four quadrupedal taxa. “**M_body_**” shows the body mass (or mean for multiple specimens). “**M_extensor_ / M_body_**” shows the percentage of whole body mass dedicated to sampled extensor muscles (one hindlimb only, and one forelimb if available). “**M_musc_ /M_body_**” shows the percentage of body mass dedicated to limb musculature (one hindlimb only, and one forelimb if available). “**M_limbs_ / M_body_**” shows the percentage of body mass that the sampled limb mass (for left and right limbs) constitutes. ^1^ Red kangaroo (*Macropus rufus*). ^2^ Bennet's wallaby (*Macropus rufogriseus*).

*Note that these data are for one individual and it is far from conclusive that, as the table might imply, emus have larger leg muscles than ostriches; it could merely be caused by individual variation and measurement error. The *Tyrannosaurus* data shown are the upper end (over)estimates for all four adult models (Tables 5,6,9), showing the range of values from minimal and maximal models. Mean and standard deviation (SD) values at bottom exclude *Tyrannosaurus*.

Our very large upper end estimates for the hip extensor muscle masses (∼9–16% body mass per limb in *Tyrannosaurus*; 2–3 times those in birds [67]), including the CFL, dominate our estimates of limb muscle masses. The distal limb muscle mass estimates from our study are far less exceptional (∼3–7% body mass for the knee; ∼2–3% for the ankle) and generally smaller (half the size or more) than those in extant ratites [67]. This is important, because Hutchinson [21], [67] noted that ankle extensor muscle masses may be the critical limit on running capacity; more so than more proximal muscles. Thus even large hip and/or knee extensor muscle masses, impressive as they may be, might not be key determinants of running ability in tyrannosaurs.

One speculative explanation for the large hip muscles in tyrannosaurs and related theropod taxa is that the hip muscles had to both retract the femur [76] and balance the cranially-positioned COM (our data and previous studies). Considering that these muscles generally should have lacked long tendons [4], [19], [54], [55], [76], [84], most length change during femoral retraction would have been in the muscle fibres. In order to achieve femoral retraction many muscles (especially uniarticular ones, effectively including the CFL) would thus have been contracting concentrically; i.e., shortening while active. It is well known that concentric activity places muscles at regions of their force-length and force-velocity curves that are disadvantageous for producing force. This disadvantage during peak power production can be as great as limiting the stress to 1/3 the maximal isometric stress [74], [104]–[107]. Power production is necessary for the task of femoral retraction (via muscle-driven hip extension), and hip extensor muscles should have generated much of the propulsive power as in many extant non-avian species [108]–[110], [114]. Thus large hip extensor muscles may have been required in non-avian theropods such as *Tyrannosaurus* to produce large moments countering the flexor moment of the COM about the hip [20], [21], [28], as well as to simultaneously retract the femur with largely concentric muscle contractions. Testing this speculation is very difficult, particularly as the detailed mechanics (stress, length changes, work, power, etc.) have never been fully measured or even quantitatively estimated in extant archosaurs, but it deserves future consideration.

The considerations noted above are ample reason for caution in interpreting the significance of our muscle mass estimates to the controversy over tyrannosaur running speeds (reviewed in [113]). Given that our lower-end muscle mass estimates match those of Hutchinson et al. [28] and our upper end estimates are interesting yet very tentative, we see no compelling justification for revising estimates of tyrannosaur speeds to be necessarily faster than the moderate range currently proposed (5–11 ms^−1^; [21], [24]). However we hope that our methods and estimates will inspire others to search for new ways to refine the methods proposed here and further illuminate this issue.

## Materials and Methods

### Specimens and data acquisition

We used varied tools including point digitizers [22], [28], LiDAR laser scanning and similar technologies [30], [111] and computed tomography of high-fidelity casts (see below) to acquire the 3D geometry of the *Tyrannosaurus* specimens listed in Table 1. The specimens were chosen because four of them (all except CM 9380) are the most complete known *Tyrannosaurus* skeletons [34], [58]. Specimen CM 9380 (approximately 11% of skeleton but representing key portions of the skull, vertebrae and especially limbs) was chosen because of its holotype status, good representation of the larger skeletal elements, and accessibility of high-resolution scans. It was LiDAR scanned by Hand et al. [111]. The mounted skeleton of FMNH PR 2081 (“Sue”) was scanned by detectives of the Chicago Police Department's forensics unit using a Leica Scanstation 2 instrument. Scans from six points at floor level and one elevated position were merged. CT scans of research grade casts of individual limb bone elements were carried out at Loyola University Medical Center on a General Electric VCT 64 slice medical scanner. Scans were conducted with a slice thickness ranging from 1–2.5 mm at a resolution of 0.6 mm×0.6 mm. A cast of the reconstructed skull that corrects for the crushing in the actual specimen was scanned at the Livonia, MI, Ford Motor Co. in a 9 mVP industrial CT scanner. Over 600 slices with a slice thickness of 2 mm and a slice spacing of 3 mm were generated to capture the skull at a pixel resolution of 0.5×0.5 mm. Post-processing was executed by Ralph Chapman of Deck & Chapman, and by Art Anderson of Virtsurf to produce .dxf format 3D images. The “Stan” and “Jane” specimens' geometry was acquired with a LiDAR scan of a cast (Stan: Manchester Museum, University of Manchester; Jane: University of Leicester, Department of Geology) using a Z&F Imager 5600i unit. The MOR specimen data were from Bates et al. [30] and Hutchinson et al. [22]. Other than noted elsewhere, no corrections for specimen distortion were taken into account in the 3D models used for our reconstructions; they were taken at face value; an assumption we discuss later. Similarly, missing or incomplete skeletal elements are commented on in the text as appropriate, and details on preserved elements for each specimen have been listed elsewhere [34].

### Model construction

Our 3D scans were then imported into graphics software [28], [30], [31] in which elliptical section “hoops” were placed around the skeletal boundaries and lofted to create a polygon mesh representing the fleshy body contours (Fig. 1). Zero-density shapes representing air spaces in the head, neck and body (i.e., torso) were then included, following anatomical landmarks as much as possible. Inevitably this modelling process involves some subjective decisions on anatomical contours far from skeletal structures (e.g., leg segment shapes). The absence of the complete gastral basket in our specimens (Figs. 2, 3, 4) also required some approximations of ventrolateral torso contours. Our methods for reconstructing the tail cross-sectional shapes based on extant saurian tails follow [31]. The software then used the assigned densities of each body segment (set at 1000 kg m^3^) to estimate the mass (in kg), centre of mass (COM) position, and inertia tensor (3×3 matrix of inertial values; e.g. [112]) for the whole body as well as its component segments. For simplicity, inertial values are not shown in this study; other studies [28], [30] had results similar to those here. The COM position was then normalized for comparison among specimens of different sizes by dividing it by femur length, total body length, gleno-acetabular distance (GAD), or pelvic limb length [31].

Once initial “preferred” models were constructed, we performed a broad sensitivity analysis of their dimensions (following [31]) by varying the fleshy boundary points of our models as iterations that we term ‘minimal mass’ (torso boundaries fit tightly to underlying skeletal boundaries; neck, limbs, and tail x0.80 dimensions of initial model; diagonal dimensions of each section hoop x0.853 to approximate a diamond, largest airspaces; [31]), ‘maximal mass’ (all radial dimensions x1.2 from the initial model – the length of the body, limbs etc. were not altered, smallest airspaces) and four alterations to particular body segment dimensions that intentionally biased the COM position toward particular maximal positions: dorsal (maximal mass models for the trunk, neck, forelimbs and caudal sections, minimal mass for the hindlimbs), ventral (vice versa), cranial (maximal mass models for neck, trunk and forelimb segments, minimal mass for caudal segment and hindlimbs), and caudal (vice versa). We did not vary head dimensions because these are reasonably constrained by anatomical landmarks, and we did not vary forelimb dimensions because they are very small proportions of mass, difficult to accurately separate from torso mass (e.g., pectoral and scapular muscles) due to their relatively small size, and not a focus of this study.

Our six extremes of model dimensions for each specimen provide a wide set of “error bars” (despite the impossibility of constructing statistically valid 95% confidence intervals) within which we would expect the actual body mass and COM to lie. Yet the reader is cautioned not to make the error of presuming that all models are equally plausible or even that any of these extremes is plausible. By initially setting our “error bars” broadly, we aim to remain within the bounds of honest, open scientific inquiry and yet still achieve our main aims.

Two investigators working closely together (VA+JM) did all model construction for two specimens (Carnegie and Sue) whereas another (KB) modelled the remaining three specimens (Stan, MOR and Jane). This provided us with an opportunity to inspect investigator biases in model reconstructions, particularly as the Carnegie, Stan and MOR specimens are of grossly similar adult or subadult (here for brevity simply termed adult; i.e., large) sizes. Although the investigators used the same basic methodologies, we consider any potential impact of subjective judgements (see below) on our results in the Discussion. Figures 2, 3, 4 show the range of models produced.

For our comparisons between *Tyrannosaurus* specimens, we focus on the minimal models, as these have the highest fidelity to what skeletal landmarks do exist and therefore maximize comparability. All other models deviate further from known data, even though they might be more plausible to varying degrees. For completeness, however, we include all results for each specimen, segment by segment, with maximal and minimal model assumptions.

Our sample size was limited to four adults and one juvenile so we could not conduct detailed studies of scaling across the entire ontogenetic spectrum [31], [37], [49]–[55]. Therefore here we simply compare two relative endpoints of tyrannosaur ontogeny in terms of quantitative estimates of body dimensions, fully cognizant that these estimates have wide margins of error (see Discussion). Indeed, quantitative estimates for unpreserved features in extinct taxa usually only allow for general qualitative conclusions to be formulated, albeit explicitly and reproducibly (e.g., [113] for discussion).

### Muscle mass analysis

We used our 3D skeletal models to conduct additional estimations of limb extensor muscle masses. First, we calculated the volumes of the major limb bones (femur, tibia, fibula and metatarsals) from the water-tight 3D bone models, then subtracted these bone volumes from the individual segment (thigh, shank and metatarsus/pes) volumes leaving a smaller volume that would have consisted of limb muscles, skin and other minor constituents (nerves, blood vessels, cartilage, etc). This is a modest refinement of the approach of Hutchinson et al. [28] who estimated extensor muscle masses using the percentages of segment mass that those muscles constitute in extant lizards, crocodylians and birds multiplied by the *Tyrannosaurus* segment masses. Additional details of our procedure are explained further below in the Results. The method was designed to overestimate muscle masses to some degree in several ways (see Results), to get upper end estimates of maximal limb muscle masses. For example, we assumed that all non-bony segment volume would be muscle.

Second, we present a new approach for estimating the mass of the large hip extensor M. caudofemoralis longus (CFL; [4], [15], [19], [114]–[115]), which Hutchinson et al. [28] simply estimated by taking 25% of the proximal tail segment volume. Our new method was originally presented by Bates et al. [116] but a similar approach was independently conceived [19], [117]. The CFL volume was estimated in Autodesk Maya (San Rafael, CA) software by drawing a smooth curve between the lateral tip of the transverse processes and the ventral tip of the chevron for each vertebra between the sacrum proximally and the transition point of the tail [114] distally, and then continuing this curve along the ventral and lateral borders of the transverse processes, centra and chevrons to form a series of complete loops (Fig. 5). These loops were then lofted to form a solid volume, which was then deformed to connect to the fourth trochanter via a small, thin extension representing the tendon. The CFL muscle mass was then calculated from this volume, assuming 1000 kg^−3^ density. This method differed slightly from another [19] in that we included a small volume immediately below the tip of the transverse processes, but also in that Persons and Currie's [19] semi-circular CFL appears to include some of the centrum (see their figure 10). Furthermore in the images from [19] the vertebrae are abstracted as squared-off and symmetrical shapes whereas our scan data were naturally curved and asymmetrical.

We tested the accuracy of our CFL muscle mass estimation method by using our method on a CT scan of an adult Australian freshwater crocodile (*Crocodylus johnstoni*; specimen from [31]). The 3D segmented skeleton (created from CT scan data of a whole cadaver by VA; [31]) was used with no information on its actual fleshy dimensions available to the user (JM), and another user (JH) measured the actual mass of the CFL muscle in that animal using dissection (electronic balance; ±0.001 kg). We then compared the estimated CFL mass to the actual mass.

We estimated muscle volumes from limb segment volumes as in [28], with slight modification. To obtain estimates of extensor muscle volume for each segment we first subtracted the bone volumes (which were small fractions; ∼5%; of the segment volumes), and then multiplied those non-bony portions of the segment volume by the percentage of mass in each segment that is dedicated to extensor muscles in extant Sauria (based on dissection data from [21], [67]). The hip extensor muscle estimate was 54% of thigh mass plus the CFL muscle mass (from this study), the knee extensor estimate was 34% of thigh mass, and the ankle extensor estimate was 47% of shank mass.

The final outputs of our limb muscle mass analysis were extensor muscle mass estimates for the hip, knee and ankle joints of individual tyrannosaur specimens. In the Discussion, we compare these with previous estimates of the extensor masses required for fast running (as in [25], [28]), but with a focus on individual variation and ontogenetic shifts in locomotor morphology and performance in *Tyrannosaurus*.

### Growth

Our 3D computational approach allows us to partially examine whether masses computed using DME fit well with masses estimated with independent models of each specimen, though the small range of overlapping specimens available to us is a limiting factor with respect to the rigor of the test. We followed Erickson et al. [39] in generating a bivariate plot of estimated body mass as a function of histologically determined age, supplementing age data for the MOR specimen from [46]. Because our reconstruction generated a wide bracket of estimated masses for each individual, we used average mass values for each specimen, except for the Sue specimen. Because of the obvious inflation in body mass caused by the distended ribcage in Sue (see below), we employed the minimum mass estimate for this analysis. This results in a growth curve with a long somatic asymptote as indicated by the presence of an External Fundamental System comprising nine growth bands in Sue [39]. Other combinations of masses between Sue and the remaining specimens produced growth curves that were incompatible with this histological observation. Horner and Padian [46] used a different protocol for determining age palaeohistologically in their sample, so we used a range of their estimates for the age of this individual (14 & 16 years; their minimum estimate of 11–12 years is at odds with the data reported for “Jane”, which was aged using the same protocol as in Erickson et al. [39]).

Next, we cubed femoral lengths (FL) for the specimens and calculated the ratio between each specimen and the cubed FL of Sue, which is set as the apex of the growth curve, following the DME protocol. Sigmoidal functions of the form *mass  =  maximum mass/1+e^a(age-b)^+5* , where *a* and *b* represent constants determined by the equation and 5 kg represents the assumed mass at hatching (age  = 0), were fitted to each data series using least squares regression in the software Prism ver. 5.1 (GraphPad Software, Inc.; La Jolla, CA). Sigmoidal growth curves were employed by Erickson et al. [39] because they represent a common vertebrate growth pattern. The constant *a* largely determines the slope of the exponential (i.e., rapid growth) phase of the curve, while *b* represents the age at which 50% of maximum body mass is reached. The relationships between the mass estimates derived from models based on scan data and the corresponding values estimated using DME were investigated in two ways. First, we examined whether the DME estimates fall within the 95% confidence interval of the growth curve that relates model-derived mass to age. Second, we employed the weighted Akaike Information Criterion (AICc) and a sum of squares F-test to determine whether separate functions (i.e., with different values for the constants a and b) fit the two curves significantly better than a single common curve.
