# Correction: A Computational Analysis of Limb and Body Dimensions in *Tyrannosaurus rex* with Implications for Locomotion, Ontogeny, and Growth

**DOI:** 10.1371/journal.pone.0097055

**Published:** 2014-05-05

**Authors:** John R. Hutchinson, Karl T. Bates, Julia Molnar, Vivian Allen, Peter J. Makovicky

Based on our plot in Figure 6, we stated that the data in our paper support the view that DME was a reasonable first approximation for modeling growth in non-avian dinosaurs. However, several inconsistencies were introduced in this plot, a fact brought to our attention by Myhrvold (2013).

Errors include using a minimum mass of 9693 kg for “Sue” instead of the published 9501 kg, and using femoral lengths published in Kilbourne and Makovicky (2010) instead of the ones in Table 3 of our paper. An important source of ambiguity came to light as we reviewed our data: the “Jane” specimen’s femur, which is broken and incomplete, was reconstructed on the cast as being 788 mm in length, whereas other studies estimate the length at 720mm (the value used in our Fig. 6).

After reviewing these mistakes and sources of uncertainty, we now judge that the data in our paper do not support our published conclusions regarding the accuracy of DME models. We no longer consider our data sufficient to permit an evaluation of how accurate this method may be, and we wish to withdraw our earlier statements on this issue (p9: the last paragraph of the results section headed 'Growth', p13: the three paragraphs in the discussion headed ‘Implications for growth rates?'; and Figure 6).
